# Exploring the Novel Susceptibility Gene Variants for Primary Open-Angle Glaucoma in East Asian Cohorts: The GLAU-GENDISK Study

**DOI:** 10.1038/s41598-019-57066-7

**Published:** 2020-01-14

**Authors:** Yong Woo Kim, Yu Jeong Kim, Hyun Sub Cheong, Yukihiro Shiga, Kazuki Hashimoto, Yong Ju Song, Seok Hwan Kim, Hyuk Jin Choi, Koji M. Nishiguchi, Yosuke Kawai, Masao Nagasaki, Toru Nakazawa, Ki Ho Park, Dong Myung Kim, Jin Wook Jeoung

**Affiliations:** 1 Department of Ophthalmology, Seoul National University College of Medicine, Seoul, Korea; 2 Department of Ophthalmology, Seoul National University Hospital, Seoul, Korea; 3 Department of Genetic Epidemiology, SNP Genetics, Inc., Seoul, Korea; 4 Department of Ophthalmic Imaging and Information Analytics, Tohoku University Graduate School of Medicine, Miyagi, Japan; 5 Department of Ophthalmology, Tohoku University Graduate School of Medicine, Miyagi, Japan; 6 Department of Ophthalmology, Chosun University College of Medicine, Gwangju, Korea; 7 Department of Ophthalmology, Seoul National University Boramae Hospital, Seoul, Korea; 8 Healthcare System Gangnam Center, Seoul National University Hospital, Seoul, Korea; 9 Department of Advanced Ophthalmic Medicine, Tohoku University Graduate School of Medicine, Miyagi, Japan; 10 Department of Integrative Genomics, Tohoku Medical Megabank Organization, Tohoku University, Miyagi, Japan; 11 Department of Human Genetics, Graduate School of Medicine, The University of Tokyo, Tokyo, Japan; 12 Graduate School of Information Sciences, Tohoku University, Miyagi, Japan; 13 Department of Retinal Disease Control, Tohoku University Graduate School of Medicine, Miyagi, Japan

**Keywords:** Optic nerve diseases, Genetics research

## Abstract

Primary open-angle glaucoma (POAG) can develop even within normal ranges of intraocular pressure, and this type of glaucoma (so-called ‘normal-tension glaucoma [NTG]’) is highly prevalent in East Asia including Korea and Japan. We conducted exome chip analysis to identify low-frequency and rare variants associated with POAG from the primary cohort (309 POAG patients and 5,400 control, all Koreans). For replication, Korean (310 POAG patients and 5,612 controls) and Japanese (565 POAG patients and 1,104 controls) cohorts were further investigated by targeted genotyping. SNP rs116121322 in *LRRC27* showed nominally significant association with POAG in the discovery cohort (OR = 29.85, *P* = 2E–06). This SNP was validated in the Korean replication cohort but only in the NTG subgroups (OR = 9.86, *P* = 0.007). Japanese replication cohort did not show significant association with POAG (*P* .00.44). However, the meta-analysis in the entire cohort revealed significant association of rs116121322 with POAG (OR_combined_ = 10.28, *P*_combined_ = 1.4E–07). The LRRC27 protein expression was confirmed from human trabecular meshwork cells. For gene-based testing, *METTL20* showed a significant association in POAG (*P*_combined_ = 0.002) and in the subgroup of NTG (*P*_combined_ = 0.02), whereas *ZNF677* were significantly associated with only in the subgroup of high-tension glaucoma (*P*_combined_ = 1.5E–06). Our findings may provide further genetic backgrounds into the pathogenesis of POAG, especially for the patients who have lower baseline intraocular pressures.

## Introduction

Glaucoma threatens the vision of more than 60 million people globally. Primary open-angle glaucoma (POAG) is the most common sub-type of glaucoma^1^, and reversing the elevation of intraocular pressure (IOP), the most prominent risk factor for the development of POAG, remains the only therapeutic target for the management of POAG^2^. However, POAG can develop even within the normal range of IOP, resulting in so-called normal-tension glaucoma (NTG) or low-pressure glaucoma^3–6^.

Previous population-based studies have shown that NTG comprises the majority of open-angle glaucoma in East Asians; in contrast, the proportion of NTG is lower in Caucasians or individuals of African descent. The proportions of NTG were 92% in the Tajimi Study (Japan)^7^, 90% in the Handan Eye Study (China)^8^, and 77% in the Namil Study (South Korea)^9^. Although the pathogenesis of NTG development is not fully understood, these geographical and ethnic differences in the prevalence of NTG imply that genetic variations may play a role.

Genome-wide association studies (GWAS) have been increasingly applied to investigate the molecular basis of POAG pathogenesis^10,11^. Multiple genes have been reported to be associated with POAG at the genome-wide level, including *CAV1/CAV2*, *ATOH7*, *TMCO1*, *CDC7-TGFBR3*, *MPP7*, *CDKN2B-AS1*, and *SIX1/SIX6*, from various populations in Europe, the United States of America, India, Japan, and China^12–20^. *CDKN2B-AS1* and *SIX1/SIX6* have been shown to be significantly associated with NTG in individuals of European ancestry^21,22^, Han Chinese population^23^, and Japanese population^15,24,25^. The latest update on the GWAS results of POAG has been described elsewhere^26^.

Given the high prevalence of NTG in East Asians, it is worth further exploring the genetic architecture associated with glaucoma risk in this ethnic group. Accordingly, in this study, we performed an exome chip analysis for POAG and the relevant gene variants have been validated in East Asian cohorts. The aims of the present study were to identify the novel genetic loci associated with POAG in East Asian populations and to investigate the difference in genetic associations according to the baseline IOP.

## Results

### Patients and control demographics

We recruited 619 patients with POAG and 11,012 healthy controls from NBK. Replication samples of 565 POAG patients and 1,104 healthy controls were further recruited from Japan. Subjects’ demographics are provided in Table 1.

**Table 1:** Demographics of Primary Open-Angle Glaucoma (POAG) Cases and Controls.

Genotyping	Primary Cohort (Korea)			Replication Cohort #1 (Korea)			Replication Cohort #2 (Japan)		
	POAG (*n* = 309)	Control (*n* = 5,400)	*P*-value	POAG (*n* = 310)	Control (*n* = 5612)	*P*-value	POAG (*n* = 565)	Control (*n* = 1104)	*P*-value
	Exome chip	Exome chip		TaqMan Assay	Exome chip		Japonica array	Japonica array	
Age, y	56.1 ± 13.6	52.6 ± 8.4	<0.001^*^	54.701 13.5	52.101 8.9	0.001^*^	64.5 ± 11.7	59.7 ± 14.1	<0.001^*^
Age range, y	21–88	39–70	—	20–83	21–84	—	35–94	35–88	—
Female, %	50.2	54.6	0.15^†^	45.2	52.4	0.015^†^	44.4	51.1	0.01^†^

POAG: primary open-angle glaucoma, HTN: hypertension, DM: diabetes mellitus. Mean ± standard deviation, *Comparison performed using Student’s t-test, ^†^Comparison performed using chi-square test.

When POAG cases were stratified by baseline IOP, 503 cases were NTG (mean baseline IOP, 15.2 ± 3.0 mmHg), and 116 cases were high-tension glaucoma (HTG) (mean baseline IOP, 24.0 ± 6.8 mmHg) from Korea, and 446 cases were NTG (mean baseline IOP, 16.1 ± 2.7 mmHg) and 119 cases were HTG (mean baseline IOP, 27.5 ± 6.9 mmHg) from Japan. There were no differences in age and an axial length between the two groups (all *P* > 0.05). However, a higher proportion of patients with NTG eyes were women, and lower central corneal thickness measurements were observed in NTG cases (Table 2). Clinical characteristics of NTG and HTG patients from each cohort are provided in Table 2.

**Table 2:** Clinical Characteristics of High-Tension Glaucoma (HTG) and Normal-Tension Glaucoma (NTG).

	Primary Cohort (Korea)			Replication Cohort #1 (Korea)			Replication Cohort #2 (Japan)		
	HTG (*n* = 55)	NTG (*n* = 254)	*P*-value	HTG (*n* = 61)	NTG (*n* = 249)	*P*-value	HTG (*n* = 119)	NTG (*n* = 446)	*P*-value
Age, y	54.1 ± 15.3	56.5 ± 13.2	0.29^*^	54.7 ± 15.6	54.7 ± 13.0	0.99^*^	64.1 ± 10.7	64.4 ± 12.0	0.78*
Female, %	**36.4**	**53.1**	**0.035**^**†**^	39.3	46.6	0.38^†^	**36.1**	**47.5**	**0.034**^**†**^
Baseline IOP, mmHg	**24.8** ± **8.1**	**15.2** ± **3.2**	<0.001^*^	**23.3** ± **5.3**	**15.2** ± **2.9**	<0.001^*^	**27.5** ± **6.9**	**16.1** ± **2.7**	<0.001*
CCT, μm	**545.0** ± **30.7**	**532.0** ± **34.3**	0.010^*^	**547.0** ± **30.4**	**534.0** ± **33.9**	0.010^*^	**520.0** ± **35.5**	**509.3** ± **34.4**	0.012*
AXL, mm	24.7 ± 1.6	24.5 ± 1.6	0.47^*^	24.9 ± 2.4	24.8 ± 1.7	0.88^*^	25.4 ± 1.8	25.2 ± 1.8	0.47*
Average RNFLT, μm	**64.0** ± **11.6**	**70.9** ± **11.4**	<0.001^*^	**63.6** ± **14.9**	**70.8** ± **12.1**	0.002^*^	76.8 ± 13.2	80.1 ± 13.9	0.051*
MD, dB	**−15.2** ± **10.1**	**−8.8** ± **6.9**	<0.001^*^	**−13.6** ± **9.0**	**−7.0** ± **6.1**	<0.001^*^	−14.8 ± 9.1	−13.1 ± 8.5	0.12*

HTG: high-tension glaucoma, NTG: normal-tension glaucoma, IOP: intraocular pressure, CCT: central corneal thickness, AXL: axial length, RNFL: retinal nerve fiber layer, MD: mean deviation. Mean ± standard deviation, *Comparison performed using Student’s t-test, ^†^Comparison performed using chi-square test.

### Single-variant associations with POAG

The minor allele frequency (MAF) distribution from exome chip analysis was highly skewed towards very low-frequency variants, with 81.3% (*n* = 198,878) of variants successfully genotyped as monomorphic and 8.1% (*n* = 119,850) as having a MAF less than 0.05, and 10.6% (*n* = 25,824) as having a MAF greater than 0.05. There was no significant stratification detected using all SNPs (Supplementary Material Fig. S1), and the genomic inflation factor (lambda) was 1.08, showing no significant dispersion of test statistics from the expected distribution (Supplementary Material Fig. S2).

**Figure 1:**
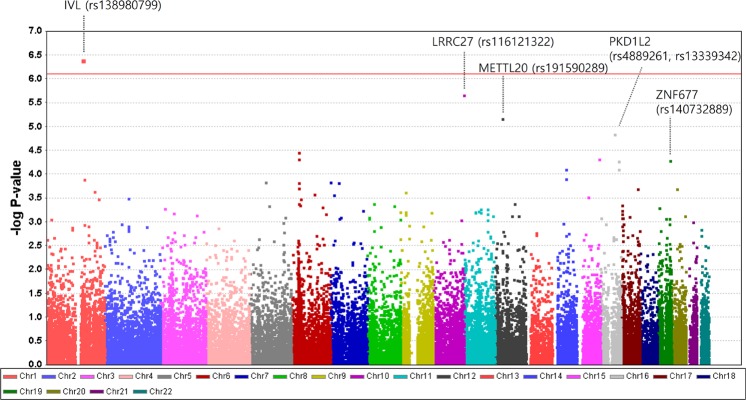
Manhattan plot of the associations of primary open-angle glaucoma from an analysis of quality control passed 63,880 single nucleotide polymorphisms on a custom HumanExome BeadChip v1.0 (Illumina, Inc.). The red line represents *P* = 7.8E–07, the level set significant after Bonferroni correction.

The strongest association for the risk of POAG was provided by rs138980799 (OR = 83.24, *P* = 3.4.0E–07) in *IVL*, which causes an H162R amino acid change, with exome-wide statistical significance. Additionally, nominally significant associations were observed for rs116121322 (OR = 29.85, *P* = 2.2E–06) in *LRRC27*, rs191590289 (OR = 3.42, *P* = 6.9E–06) in *METTL20*, rs140732889 (OR = 20.72, *P* = 5.1E–05) in *ZNF677*, and rs4889261 (OR = 0.33, *P* = 5.4E–05) and rs13339342 (OR = 0.34, *P* = 37.8E–05) in *PKDIL2*; however these associations did not reach statistical significance after Bonferroni corrections for multiple testing (Table 3, Supplementary Material Table S1, and Fig. 1).

**Table 3:** Association of rs116121322 in *LRRC27* with Primary Open-Angle Glaucoma.

rsID	Gene	Chr	AA change	Alleles	Population	Stage	MAF Case	MAF Control	OR (95% CI)	*P*-value
rs116121322	LRRC27	10	V189I	G > A	POAG	Primary (KOR)	**0.00971**	**0.00037**	**29.85 (7.99–111.59)**	**2.2E-06**
						Replication#1 (KOR)	**0.00647**	**0.00054**	**10.40 (2.82–38.32)**	**0.002**
						Replication#2 (JPN)	0.00090	0.00241	0.43 (0.05–3.78)	0.44
						Combined (KOR)	**0.00809**	**0.00046**	**17.79(7.19**–**44.01)**	1.6E-07^*^
						Meta-analysis (KOR + JPN)			**10.28**	1.4E-07^*^
					NTG	Primary (KOR)	**0.00787**	**0.00037**	**18.75 (4.47**–**78.69)**	**0.0004**
						Replication#1 (KOR)	**0.00604**	**0.00054**	**9.86 (2.35**–**41.31)**	**0.007**
						Replication#2 (JPN)	0.00135	0.00241	0.67 (0.08-5.88)	0.72
						Combined (KOR)	**0.00697**	**0.00046**	**14.13 (5.21**–**38.37)**	2.3E-05^*^
					HTG	Primary (KOR)	**0.01818**	**0.00037**	**76.78 (13.32**–**442.58)**	0.0004
						Replication#1 (KOR)	**0.00820**	**0.00054**	15.18 (1.77–130.02)	0.06
						Replication#2 (JPN)	0.00000	0.00241	NA	NA
						Combined (KOR)	**0.01293**	**0.00046**	**28.91 (7.85**–**106.46)**	3.8E-04^*^

Chr: chromosome, AA: amino acid, MAF: minor allele frequency, POAG: primary open-angle glaucoma (i.e., NTG OAHTG), NTG: normal-tension glaucoma, HTG: high-tension glaucoma, OR: odds ratio, KOR: Korea, JPN: Japan, NA: not applicable. ^*^P-values adjusted by Benjamini-Hochberg method to compensate for multiple comparison.

The 6 SNPs mentioned above were further validated from the replication cohort #1 (Korea) and cohort #2 (Japan). In replication cohort #1 (Korea), only the SNP rs116121322 in *LRRC27*, which causes a V189I amino acid change, revealed a significant association with POAG (OR = 10.40, *P* = 0.002). However, this SNP did not show any statistical significance in replication cohort #2 (Japan) (OR = 0.43, *P* = 0.44). The combined OR in Korean population was 17.79 (adjusted *P* = 1.6E–07), and the meta-analysis with the Japanese cohort still exhibited a significant association with POAG (OR = 10.28, adjusted *P* = 1.4E–07, Table 3).

The SNPs rs138980799 in *IVL*, rs191590289 in *METTL20*, rs140732889 in *ZNF677*, and rs4889261 and rs13339342 in *PKDIL2* did not reach statistical significance with POAG from both replication cohort #1 (Korea) and cohort #2 (Japan). However, the combined analysis from Korean population showed significant association with POAG from the SNPs rs138980799 in *IVL* (OR_combined_ = 27.40, adjusted *P* = 1.6E–05), rs191590289 in *METTL20* (OR_combined_ = 1.83, adjusted *P* = 0.012), and rs140732889 in *ZNF677* (OR_combined_ = 9.60, adjusted *P* = 0.001, Supplementary Material Table S1).

### Single-variant association differences between patients with NTG and HTG

The rs116121322 in *LRRC27* was associated with NTG in primary cohort and replication cohort #1 (Korea) but not in replication cohort #2 (Japan). However, the combined analysis in entire Korean cohorts showed a significant association with NTG (OR_combined_ = 14.13, adjusted *P* = 2.3E–05, Table 3). The SNPs rs138980799 in *IVL* and rs191590289 in *METTL20* were associated with NTG in the primary cohort and from a combined analysis of entire Korean cohorts but found to be monomorphic in replication cohort #2 (Supplementary Material Table S2). The significant findings of SNPs rs4889261 and rs13339342 in PKD1L2 from exome chip analysis did not reach statistical significance in validation analysis from replication cohorts #1 and #2 (Supplementary Material Table S2).

The SNPs rs116121322 in *LRRC27* (OR_combined_ = 28.91, adjusted *P* = 3.8E–04) and rs140732889 in *ZNF677* (OR_combined_ = 39.93, adjusted *P* = 3.8E–05) showed significant association with HTG from primary cohort and combined analysis form entire Korean cohorts (Table 3 and Supplementary Material Table S2). The rs191590289 in *METTL20* was significantly associated with HTG only in primary cohort (OR = 4.85, *P* = 0.007) but not in replication cohorts. The SNPs rs4889261 (OR_combined_ = 0.35, adjusted *P* = 0.013) and rs13339342 (OR_combined_ = 0.36, adjusted *P* = 0.013) in *PKD1L2* showed only marginal significance from combined analysis of entire Korean cohorts (Supplementary Material Table S2).

### Gene-based association analysis

Since the majority of individual variants are very rare (median MAF = 0.0084), we assessed the burden of 63,880 variants across 13,923 genes. *METTL20* (N172S, D188N, and D194V) showed an exome-wide significant association with POAG (adjusted *P* = 0.006) from the primary cohort but did not reach statistical significance in replication cohort #1. The combined analysis showed a significant association with POAG (*P* = 0.002). This gene revealed a significant association with NTG from entire Korean cohorts (*P* = 0.02) but not in HTG eyes. *ZNF677* (G180R and Y347T) was significantly associated with HTG eyes but not with NTG eyes, with exome-wide statistical significance from the primary cohort (adjusted *P* = 3.6E–05) and in entire Korean cohorts (*P* = 1.5E–06) (Supplementary Material Table S3).

### LRRC27 Protein expression in human trabecular meshwork cells

**Figure 2:**
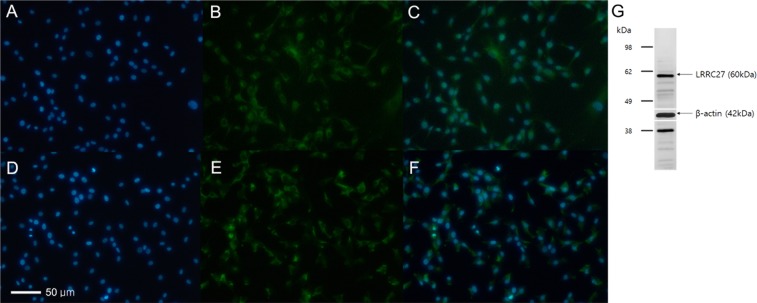
Expression of LRRC27 protein from human trabecular meshwork cells (HTMC). (**A**) DAPI, (**B**) LRRC27, (**C**) Merged image of (**A**,**B**,**D**) DAPI, (**E**) α-SMA, the marker for HTMC (positive control), (**F**) Merged image of (**D**,**E**,**G**) Western blot analysis demonstrated expression of LRRC27 protein (60 kDa). The β-actin was used as positive control. The blots from LRRC27 and β-actin were cropped from different gels and grouped together (Cropped region marked as white line). Full-length blots/gels are presented in Supplementary Material Fig. S3.

To determine whether *LRRC27* gene is a candidate for involvement in the pathogenesis of glaucoma, the expression of LRRC27 protein from human trabecular meshwork cells (HTMC) was confirmed by western blot and immunofluorescence analysis (Fig. 2). The LRRC27 proteins were confirmed to be expressed at the cytosol of HTMCs.

## Discussion

In this study, we performed an exome-wide association study for POAG in a Korean population, and reviewed their results from Japanese population, in which the prevalence of NTG is very high compared with that in other regions or races. Our data identified one novel candidate gene variant (rs116121322 in *LRRC27*) associated with the risk of POAG, which revealed statistical significance from Korean population and meta-analysis from Korean and Japanese population.

The SNP rs116121322 in *LRRC27* (encoding leucine-rich repeat-containing protein 27) was significantly associated with POAG. The SNP rs116121322 is a missense variant or non-coding transcript variant; however its clinical significance has not yet been clearly elucidated. The gene *LRRC27* has 14 transcripts, of which 5 are containing an open reading frame. The cDNA of *LRRC27* is expressed in various tissues, including the fetal eye, lens, anterior segment, optic nerve, and retina, while the level of expression is highest from sex organs (testis followed by fallopian tube)^27,28^. As the cDNA of *LRRC27* been confirmed to be expressed in ocular tissues including optic nerve and retina, the gene variant may be expected to alter the physiology of the optic nerve. The present data further confirmed the expression of LRRC27 protein from HTMCs. From the findings that this gene has a high expression of mRNA at sex organs, altered metabolism of sex-hormones or altered sex organ-related functions may have increased the risk for glaucoma development. Evidence is increasing that the retina and optic nerve are sex hormone-sensitive tissues^29–31^. In addition, epidemiological studies reveal higher prevalence of glaucoma in men than women^1,32,33^. Interestingly, MAF of rs116121322 for POAG and healthy control differed by gender in our cohorts (male, 0.62% vs. 0.05%, female, 1.02% vs. 0.04%) (Supplementary Material Table S4). Further functional investigation to explore the role of this gene variant on optic nerve head tissues and its relationship with gender is needed. Unlike expectations, this SNP was not associated with POAG in Japanese cohorts. Considering the low MAF of rs116121322, relatively small population size of healthy controls of Japan than those of Korea (1,104 vs. 11,012) may have biased the association results.

In this study, the SNP rs138980799 in *IVL* (involucrin) was significantly associated with NTG, although failed to be validated from the replication cohorts. The *IVL* gene encodes a protein, involucrin, that is expressed in keratinocytes in the epidermis and other stratified squamous epithelia, including the cornea^34^. This protein is synthesized during terminal differentiation and becomes cross-linked to membrane proteins through the catalytic function of transglutaminase, contributing to the formation of a cell envelope that protects corneocytes^35^. Involucrin expression is known to be altered in response to environmental biomechanical changes, i.e., when corneal keratinocytes are exposed to nonphysiological substrate elasticity^36^. Considering the close relationship between corneal biomechanics and POAG development and/or progression, our results provide insights into the role of involucrin in the pathogenesis of POAG.

Gene-based analysis revealed that the combination of SNPs in *METTL20* was significantly associated with NTG but not with HTG. METTL20 is a mitochondrial lysine-specific methyltransferase that targets the β subunit of electron transfer flavoprotein (ETF) and modulates its activity^37^. ETF transfers electrons to the ubiquinone pool of the mitochondrial respiratory chain via quinone oxidoreductase. Alterations in METTL20 expression may impair the mitochondrial respiratory chain and induce oxidative stress, which is strongly implicated in the pathogenesis of POAG^38,39^. Recently, our group reported a spectrum of mitochondrial DNA variants in a patient with NTG^40^. The current study may further support that mitochondrial dysfunction may be a genetic risk factor for the development of POAG.

The SNP rs140732889 in *ZNF677* (encoding zinc finger protein 677) was significantly associated with HTG but not with NTG from the exome-chip analysis. Gene-based analysis showed consistent results with a combination of variants in *ZNF677*. The *ZNF677* gene is a tumor suppressor in non-small cell lung cancers; however, the clinical implications of the SNP rs140732889 have not yet been reported^41^. Tumor-suppressor genes are often associated with the pathogenesis of POAG. For example, polymorphisms in the *TP53* gene, a prototypical tumor-suppressor gene encoding a 53-kDa protein (p53), have been shown to be associated with POAG^42^. The *INK4* locus at chromosome 9p21, which encodes three tumor-suppressor genes (*CDKN2A*, *ARF*, and *CDKN2B*), has been reported to be associated with POAG and with retinal ganglion cell (RGC) susceptibility in mice^43,44^. Given that POAG is characterized by dysregulation of RGC apoptosis, the role of tumor-suppressor genes in POAG pathogenesis should be investigated further in the clinical setting.

Our current findings revealed that the SNPs rs4889261 and rs13339342 in *PKD1L2* (encoding polycystic kidney disease 1-like 2) were marginally associated with HTG. This gene encodes a member of the polycystin protein family, which includes polycystin-1 (PC1). Aberrant expression of this gene is known to cause autosomal dominant polycystic kidney disease (ADPKD)^45^. PC1 localizes to the apical primary cilia and interacts with polycystin-2 (PC2) to form a receptor complex that transduces Ca^2+^ signals in response to renal flow and maintains the cilia in a differentiated state^46–48^. Dysfunctions in primary cilia signaling can give rise to retinal degeneration, including Bardet-Biedl syndrome or Lowe syndrome^49–51^. Recently, Luo *et al*.^52^. demonstrated that the primary cilia in trabecular meshwork cells respond to pressure changes and mediate subsequent signal transduction to regulate the aqueous humor balance. Thus, the PC1 dysfunction may have deteriorated the ability of primary cilia to sense the IOP, resulting in impaired signal transduction.

The present study has several limitations. First, relatively small POAG population may have weakened the statistical power to detect associations of low-frequency variants. This may have precluded identifying significant associations from our study population. To overcome this limitation, gene-based analysis, the SKAT-O method, was used to reduce the number of tests performed and enhance the detection ability for low-frequency variants. Second, the design of the exome chip was based on pooled exome sequencing data, in which the majority of included individuals were European Americans. These data may have lacked information regarding some important rare alleles in Asian populations.

In conclusion, the present study, for the first time, identified novel low-frequency variants associated with POAG risk in East Asian population by exome chip analysis. The SNP in *LRRC27* gene was significantly associated with POAG and confirmed to be expressed in HTMC. Gene-based testing demonstrated *METTL20* gene to be associated with NTG while ZNF677 gene is associated with HTG. Our current findings may provide further genetic and pathophysiological pathways for understanding the pathogenesis of POAG.

## Materials and Methods

This study was undertaken as a part of the GLAU-GENDISK (GLAUcoma GENe DIscovery Study in Korea) project, which is an ongoing prospective study designed in 2011. The primary objective of the GLAU-GENDISK project was to investigate and identify novel genetic susceptibility loci for various types of glaucoma in a Korean population. The secondary objectives included establishing the genotype-phenotype relationships in glaucoma patients and constructing new disease prediction models. The Japanese POAG patients were further recruited from the institutes related to Tohoku University and the control subjects were recruited from the Tohoku Medical Megabank Organization.

The present study was approved by the Seoul National University Hospital Institutional Review Board and followed the tenets of the Declaration of Helsinki (1964). Written informed consent was obtained from each of the enrolled participants. The Institutional Review Board of the Tohoku Graduate School of Medicine approved the secondary use of the genomic array data. All methods were performed in accordance with the relevant guidelines and regulations.

### Study population

All subjects included in this analysis were of Asian descent. The participants in this study included 622 patients with POAG and 213 healthy controls who were enrolled in the GLAU-GENDISK, 10,799 healthy controls from the population-based cohorts in the Korean Genome and Epidemiology Study (KoGES), and 565 patients with POAG and 1,104 healthy controls from Japan. Patients with POAG in Korea were recruited from Seoul National University Hospital, Seoul National University Boramae Hospital, and Healthcare System Gangnam Center in Korea. The data for healthy Korean controls were further provided by the National Biobank of Korea (NBK). The NBK secured biospecimens from the general population from various cohorts organized by the Korea National Institute of Health (KNIH)^53^. Exome chip analysis data and epidemiological survey data from each participant (including lifestyle, medical history, physical activity, food consumption, disease-related blood test results, and body measurements) were obtained from healthy populations from the population-based cohorts in the KoGES, including the KoGES_Ansan and Ansung study and the KoGES_health examinee (HEXA) study^53^. All participants were proven to have healthy eyes based on the survey.

The Korean POAG patients and healthy controls were randomly classified to the primary cohort (312 POAG patients and 5,400 controls) and replication cohort #1 (310 POAG patients and 5,612 controls). The Japanese POAG patients and healthy controls were assigned as replication cohort #2.

POAG was defined as the presence of glaucomatous optic disc changes with corresponding glaucomatous visual field (VF) defects and an open angle confirmed by gonioscopic examination. Glaucomatous optic disc changes were defined as neuroretinal rim thinning, notching, excavation, or retinal nerve fiber layer (RNFL) defects. Glaucomatous VF defects were defined as (1) glaucoma hemifield test values outside the normal limits, (2) three or more abnormal points with a probability of being normal of *P* < 5%, of which at least one point has a pattern deviation of *P* < 1%, or (3) a pattern standard deviation of *P* < 5%. The VF defects were confirmed on two consecutive reliable tests (fixation loss rate ≤20%, false-positive and false-negative error rates ≤25%). The baseline IOP value was defined as the mean of at least two measurements before initiation of IOP-lowering treatment. Based on the baseline IOP values, HTG eyes were defined as POAG eyes with a baseline IOP of greater than 21 mmHg, and NTG eyes were defined as POAG eyes with baseline IOP of less than or equal to 21 mmHg.

Patients with POAG in GLAU-GENDISK cohort underwent a complete ophthalmic examination, including a visual acuity assessment, slit-lamp biomicroscopy, gonioscopy, Goldmann applanation tonometry, refractions, dilated fundus examination, disc stereophotography, and red-free fundus photography using a digital fundus camera (VX-10; Kowa, Nagoya, Japan) and standard automated perimetry (Humphrey C 24-2 SITA-Standard visual field; Carl Zeiss Meditec, Inc., Dublin, CA, USA). The central corneal thickness (Pocket II; Quantel Medical, Clermont-Ferrand, France) and axial length (AXIS-II Ultrasonic Biometer; Quantel Medical S.A., Bozeman, MT, USA) were measured. A 200 × 200 optic disc cube scan was performed using Cirrus HD-OCT (Carl-Zeiss Meditec), and the average peripapillary RNFL thickness was measured with the built-in analysis algorithm (software version 6.0; Carl Zeiss Meditec). A 200 × 200 macular cube scan was performed to obtain the macular ganglion cell-inner plexiform layer (GCIPL) thickness.

POAG patients from Japan also underwent a complete ophthalmic examination including a visual acuity assessment, slit-lamp biomicroscopy, gonioscopy, Goldmann applanation tonometry, refractions, stereoscopic fundus camera photographs (nonmyd WX, Kowa Company, Nagoya, Japan), central corneal thickness (CASIA, Tomey Cooperation, Nagoya, Japan), axial length (IOLMaster, Carl Zeiss Meditec), standard automated perimetry (Humphrey C 24-2 SITA-Standard visual field; Carl Zeiss Meditec), and OCT scan (3D OCT 2000, Topcon, Tokyo, Japan)^25^.

The present study excluded participants with a diagnosis or history of any secondary glaucoma, a history of ocular trauma, a history of systemic or ocular infection, or a history of systemic or ocular use of glucocorticoids.

### Exome chip analysis

Study samples from primary cohort were processed on a HumanExome Bead-Chip 12v1-1 system (Illumina, Inc.; San Diego, CA, USA), which included 244,651 markers focused on protein-altering variants. Details regarding SNP content and selection strategies can be found at the exome array design webpage (http://genome.sph.umich.edu/wiki/Exome_Chip_Design). Genotype calling was performed using Illumina’s GenTrain version 2.0 clustering algorithm with GenomeStudio software (V2011.1). Cluster boundaries were determined using Illumina’s standard cluster file. After additional visual inspection of SNPs with call rates of less than 0.99 and SNPs with minor allele frequencies of less than 0.002, 244,552 of 244,651 (99.96%) attempted markers were successfully genotyped with call rates greater than 98% (average call rate: 99.92%). In total, 309 of 312 patients were successfully genotyped (call rate >98%).

The obtained control dataset from NBK, processed with the HumanExome Bead-Chip 12v1-1 system, passed the quality control criteria (call rate >98%). Individuals who had POAG or NTG were excluded, as were related individuals whose estimated identity-by-state values were high (>0.50). We carried out principal component analysis (PCA) to avoid artifactual results due to family relatedness. The possible population stratification in this study using PCA was examined using HelixTree.

After excluding monomorphic SNPs, 63,880 SNPs were used for statistical analysis. SNP genotype frequencies were examined for Hardy-Weinberg equilibrium using the chi-squared statistics and all were found to be consistent (*P* > 0.05). Data were analyzed using an unconditional logistic regression to calculate an odds ratio (OR) as an estimate of the relative risk of POAG associated with SNP genotypes. To determine the association between the genotype and haplotype distributions, a logistic analysis was performed controlling for age and sex as covariates to eliminate or reduce any confounding factors that could influence the findings.

For gene-based testing, we used the SKAT-O test^54^, which encompassed burden tests and SKAT as special cases^55^. SKAT-O has been shown to perform well under a range of scenarios, including scenarios in which protective, deleterious, and null variants are present and those in which a large number of variants are causal and associated in the same direction^54^.

### Validation analysis

Targeted genotyping of 6 SNPs (rs116121322 in *LRRC27*, rs138980799 in *IVL*, rs191590289 in *METTL20*, rs140732889 in *ZNF677*, and rs4889261 and rs13339342 in *PKD1L2*) in the POAG samples from replication cohort #1 was carried out by the Taqman assay (Applied Biosystems, Carlsbad, CA, USA)^56^. The Taqman assay was performed according to following steps: (1) preparation of approximately 20 ng of purified genomic DNA; and (2) preparation of genotyping mixture consisting of 2X genotyping master mix, 20X SNP genotyping assay, DNAse-free water and template DNA; and (3) polymerase chain reaction (PCR) containing 40 cycles of denaturation and annealing/extension steps^56^. When completed PCR, genotypes of DNA samples were analyzed on the ABI prism 7900HT sequence detection system (Applied Biosystems, Foster City, CA). Genotyping quality control was performed in 10% of the samples by duplicate checking (rate of concordance in duplicates >99.5%) The call rate was 99.5% in all of the 6 SNPs and was therefore considered informative. Study samples from replication cohort #2 were processed on a Japonica array (Toshiba, Tokyo, Japan), a custom-designed array optimized for the Japanese population based on the information from the reference panel from 1,070 Japanese^25^. SNP quality control and the imputation procedure were performed by using a 1,070 Japanese whole-genome panel as previously reported^24^. For meta-analysis of datasets from Korea and Japan, the basic meta-analysis function in PLINK was applied. Fixed-effect meta-analysis *P* value and fixed-effect OR were estimated.

### Cell culture

Human trabecular meshwork cells (HTMC) were purchased from Sciencell Research Laboratories (San Diego, CA, USA) and cultured in trabecular meshwork cell medium (Sciencell). The cells were passaged by trypsinization every 3–4 days.

### Western blot analysis

For Western blot analysis, the collected HTMCs lysed using a RIPA buffer with protease inhibitor cocktail (Sigma, St Louis, MO, USA). Protein extracts were separated on 10% SDS-PAGE and transferred to polyvinylidene difluoride (PVDF) membranes. The membranes were blocked with 5% nonfat dried milk and incubated overnight with LRRC27 rabbit polyclonal antibody (1:500, Novus Biologicals, Littleton, USA) or β-actin mouse monoclonal antibody (1:500, Sigma) at 4 °C. Then the membranes were washed and incubated with mouse anti-rabbit IgG or goat anti-mouse IgG (1:1000; Life Technologies, Eugene, OR, USA) for 1 hour. The immunoreactive bands were detected by chemiluminescent horseradish peroxidase (HRP) substrates (Thermo Scientific, Rockford, USA).

### Immunofluorescence

HTMCs were fixed in 10% neutral buffered formalin for 10 minutes and washed with phosphate-buffered saline (PBS). Cells were blocked with bovine serum albumin (BSA) solution for 30 minutes and incubated with primary antibody overnight at 4 °C. After washing with PBS, cells were incubated with Alexa Fluor 488-conjugated anti-rabbit (Molecular Probes, Eugene, OR, USA) and counterstained with 4′,6-diamidino-2-phenylindole (DAPI, Sigma). Stained cells were examined by fluorescence microscope (DMI4000B; Leica, Germany).

## Supplementary information

Supplementary Data.

## Data Availability

The datasets generated during and/or analysed during the current study are available from the corresponding author on reasonable request. The microarray sequencing dataset is held at Seoul National University Hospital (SNUH), and access to the dataset needs approval of Institute of Review Board of SNUH.

## References

[1] Tham YC (2014). Global prevalence of glaucoma and projections of glaucoma burden through 2040: a systematic review and meta-analysis. Ophthalmology.

[2] Kwon YH, Fingert JH, Kuehn MH, Alward WL (2009). Primary open-angle glaucoma. N. Engl. J. Med..

[3] The effectiveness of intraocular pressure reduction in the treatment of normal-tension glaucoma. Collaborative Normal-Tension Glaucoma Study Group. *American journal of ophthalmology* **126**, 498–505 (1998). 10.1016/s0002-9394(98)00272-4 9780094

[4] Comparison of glaucomatous progression between untreated patients with normal-tension glaucoma and patients with therapeutically reduced intraocular pressures. Collaborative Normal-Tension Glaucoma Study Group. *American journal of ophthalmology* **126**, 487–497 (1998). 10.1016/s0002-9394(98)00223-2 9780093

[5] Anderson DR, Drance SM, Schulzer M, Collaborative Normal-Tension Glaucoma Study, G. (2001). Natural history of normal-tension glaucoma. Ophthalmology.

[6] Drance S, Anderson DR, Schulzer M, Collaborative Normal-Tension Glaucoma Study, G. (2001). Risk factors for progression of visual field abnormalities in normal-tension glaucoma. Am. J. Ophthalmol..

[7] Iwase A (2004). The prevalence of primary open-angle glaucoma in Japanese: the Tajimi Study. Ophthalmology.

[8] Liang YB (2011). Prevalence of primary open angle glaucoma in a rural adult Chinese population: the Handan eye study. Invest. Ophthalmol. Vis. Sci..

[9] Kim CS, Seong GJ, Lee NH, Song KC, Namil Study Group, K. G. S. (2011). Prevalence of primary open-angle glaucoma in central South Korea the Namil study. Ophthalmology.

[10] Mackey DA, Hewitt AW (2014). Genome-wide association study success in ophthalmology. Curr. Opin. Ophthalmol..

[11] Abu-Amero K, Kondkar AA, Chalam KV (2015). An Updated Review on the Genetics of Primary Open Angle Glaucoma. Int. J. Mol. Sci..

[12] Thorleifsson G (2010). Common variants near CAV1 and CAV2 are associated with primary open-angle glaucoma. Nat. Genet..

[13] Burdon KP (2011). Genome-wide association study identifies susceptibility loci for open angle glaucoma at TMCO1 and CDKN2B-AS1. Nat. Genet..

[14] Osman W, Low SK, Takahashi A, Kubo M, Nakamura Y (2012). A genome-wide association study in the Japanese population confirms 9p21 and 14q23 as susceptibility loci for primary open angle glaucoma. Hum. Mol. Genet..

[15] Nakano M (2012). Common variants in CDKN2B-AS1 associated with optic-nerve vulnerability of glaucoma identified by genome-wide association studies in Japanese. PLoS One.

[16] Li Z (2015). A common variant near TGFBR3 is associated with primary open angle glaucoma. Hum. Mol. Genet..

[17] van Koolwijk LM (2012). Common genetic determinants of intraocular pressure and primary open-angle glaucoma. PLoS Genet..

[18] Ramdas WD (2011). Common genetic variants associated with open-angle glaucoma. Hum. Mol. Genet..

[19] Iglesias AI (2014). Exome sequencing and functional analyses suggest that SIX6 is a gene involved in an altered proliferation-differentiation balance early in life and optic nerve degeneration at old age. Hum. Mol. Genet..

[20] Vishal M (2016). Genetic association and stress mediated down-regulation in trabecular meshwork implicates MPP7 as a novel candidate gene in primary open angle glaucoma. BMC Med. Genomics.

[21] Mabuchi F (2012). Association between genetic variants associated with vertical cup-to-disc ratio and phenotypic features of primary open-angle glaucoma. Ophthalmology.

[22] Wiggs JL (2012). Common variants at 9p21 and 8q22 are associated with increased susceptibility to optic nerve degeneration in glaucoma. PLoS Genet..

[23] Chen Y (2015). Genetic Variants Associated With Different Risks for High Tension Glaucoma and Normal Tension Glaucoma in a Chinese Population. Invest. Ophthalmol. Vis. Sci..

[24] Takamoto M (2012). Common variants on chromosome 9p21 are associated with normal tension glaucoma. PLoS One.

[25] Shiga Y (2017). Genetic analysis of Japanese primary open-angle glaucoma patients and clinical characterization of risk alleles near CDKN2B-AS1, SIX6 and GAS7. PLoS One.

[26] Youngblood H, Hauser MA, Liu Y (2019). Update on the genetics of primary open-angle glaucoma. Exp. eye Res..

[27] Sayers EW (2009). Database resources of the National Center for Biotechnology Information. Nucleic Acids Res..

[28] Benson DA, Karsch-Mizrachi I, Lipman DJ, Ostell J, Sayers EW (2009). GenBank. Nucleic Acids Res..

[29] Munaut C (2001). Presence of oestrogen receptor type beta in human retina. Br. J. Ophthalmol..

[30] Akar ME, Taskin O, Yucel I, Akar Y (2004). The effect of the menstrual cycle on optic nerve head analysis in healthy women. Acta ophthalmologica Scandinavica.

[31] Agapova OA, Kaufman PL, Hernandez MR (2006). Androgen receptor and NFkB expression in human normal and glaucomatous optic nerve head astrocytes *in vitro* and in experimental glaucoma. Exp. eye Res..

[32] Rudnicka AR, Mt-Isa S, Owen CG, Cook DG, Ashby D (2006). Variations in primary open-angle glaucoma prevalence by age, gender, and race: a Bayesian meta-analysis. Invest. Ophthalmol. Vis. Sci..

[33] Kapetanakis VV (2016). Global variations and time trends in the prevalence of primary open angle glaucoma (POAG): a systematic review and meta-analysis. Br. J. Ophthalmol..

[34] Eckert RL (2004). Regulation of involucrin gene expression. J. Investig. dermatology.

[35] Kalinin AE, Kajava AV, Steinert PM (2002). Epithelial barrier function: assembly and structural features of the cornified cell envelope. Bioessays.

[36] Moers K (2013). Substrate elasticity as biomechanical modulator of tissue homeostatic parameters in corneal keratinocytes. Exp. Cell Res..

[37] Malecki J, Ho AY, Moen A, Dahl HA, Falnes PO (2015). Human METTL20 is a mitochondrial lysine methyltransferase that targets the beta subunit of electron transfer flavoprotein (ETFbeta) and modulates its activity. J. Biol. Chem..

[38] Van Bergen NJ (2015). Measurement of Systemic Mitochondrial Function in Advanced Primary Open-Angle Glaucoma and Leber Hereditary Optic Neuropathy. PLoS One.

[39] Osborne NN, Nunez-Alvarez C, Joglar B, Del Olmo-Aguado S (2016). Glaucoma: Focus on mitochondria in relation to pathogenesis and neuroprotection. Eur. J. pharmacology.

[40] Jeoung JW (2014). Mitochondrial DNA variant discovery in normal-tension glaucoma patients by next-generation sequencing. Invest. Ophthalmol. Vis. Sci..

[41] Heller G (2015). DNA methylation transcriptionally regulates the putative tumor cell growth suppressor ZNF677 in non-small cell lung cancers. Oncotarget.

[42] Guo Y (2012). Association of TP53 polymorphisms with primary open-angle glaucoma: a meta-analysis. Invest. Ophthalmol. Vis. Sci..

[43] Vishal M (2014). Evaluation of genetic association of the INK4 locus with primary open angle glaucoma in East Indian population. Sci. Rep..

[44] Gao S, Jakobs TC (2016). Mice Homozygous for a Deletion in the Glaucoma Susceptibility Locus INK4 Show Increased Vulnerability of Retinal Ganglion Cells to Elevated Intraocular Pressure. Am. J. Pathol..

[45] Wilson PD (2004). Polycystic kidney disease. N. Engl. J. Med..

[46] Nauli SM (2006). Loss of polycystin-1 in human cyst-lining epithelia leads to ciliary dysfunction. J. Am. Soc. Nephrology: JASN.

[47] Wilson PD (2008). Mouse models of polycystic kidney disease. Curr. Top. Dev. Biol..

[48] Xu C (2007). Human ADPKD primary cyst epithelial cells with a novel, single codon deletion in the PKD1 gene exhibit defective ciliary polycystin localization and loss of flow-induced Ca^2+^ signaling. Am. J. Physiol. Ren. physiology.

[49] Nachury MV (2007). A core complex of BBS proteins cooperates with the GTPase Rab8 to promote ciliary membrane biogenesis. Cell.

[50] Zhang X, Jefferson AB, Auethavekiat V, Majerus PW (1995). The protein deficient in Lowe syndrome is a phosphatidylinositol-4,5-bisphosphate 5-phosphatase. Proc. Natl Acad. Sci. USA.

[51] Luo N (2013). Compensatory Role of Inositol 5-Phosphatase INPP5B to OCRL in Primary Cilia Formation in Oculocerebrorenal Syndrome of Lowe. PLoS One.

[52] Luo N (2014). Primary cilia signaling mediates intraocular pressure sensation. Proc. Natl Acad. Sci. USA.

[53] Kim Yeonjung, Han Bok-Ghee (2016). Cohort Profile: The Korean Genome and Epidemiology Study (KoGES) Consortium. International Journal of Epidemiology.

[54] Lee S, Wu MC, Lin X (2012). Optimal tests for rare variant effects in sequencing association studies. Biostatistics.

[55] Wu MC (2011). Rare-variant association testing for sequencing data with the sequence kernel association test. Am. J. Hum. Genet..

[56] Livak KJ (1999). Allelic discrimination using fluorogenic probes and the 5′ nuclease assay. Genet. Anal..

