# Supplementary material for: Exploring the Novel Susceptibility Gene Variants for Primary Open-Angle Glaucoma in East Asian Cohorts: The GLAU-GENDISK Study
Source: Sci Rep. 2020 Jan 14;10:221. doi: 10.1038/s41598-019-57066-7 (PMC6959350; doi:10.1038/s41598-019-57066-7)
Supplement: Supplementary file 1 — Supplementary Data. [file 41598_2019_57066_MOESM1_ESM.pdf]

Supplementary Information

Exploring the Novel Susceptibility Gene Variants for Primary Open-Angle Glaucoma in East Asian Cohorts: The GLAU-GENDISK Study

Yong Woo Kim, MD[1,2], Yu Jeong Kim, MS[1], Hyun Sub Cheong, PhD[3], Yukihiro Shiga, MD, PhD[4,5], Kazuki Hashimoto, MD[5], Yong Ju Song, MD[6], Seok Hwan Kim, MD, PhD[1,7], Hyuk Jin Choi, MD, PhD[1,8], Koji M Nishiguchi, MD, PhD[9], Yosuke Kawai, PhD[10,11], Masao Nagasaki, PhD[10,12], Toru Nakazawa, MD, PhD[4,5,9,13], Ki Ho Park, MD, PhD[1,2], Dong Myung Kim, MD, PhD[1], and Jin Wook Jeoung, MD, PhD[1,2]

[1] Department of Ophthalmology, Seoul National University College of Medicine, Seoul, Korea
[2] Department of Ophthalmology, Seoul National University Hospital, Seoul, Korea
[3] Department of Genetic Epidemiology, SNP Genetics, Inc., Seoul, Korea
[4] Department of Ophthalmic Imaging and Information Analytics, Tohoku University Graduate School of Medicine, Miyagi, Japan

[5] Department of Ophthalmology, Tohoku University Graduate School of Medicine, Miyagi, Japan

[6] Department of Ophthalmology, Chosun University College of Medicine, Gwangju, Korea
[7] Department of Ophthalmology, Seoul National University Boramae Hospital, Seoul, Korea
[8] Healthcare System Gangnam Center, Seoul National University Hospital, Seoul, Korea
[9] Department of Advanced Ophthalmic Medicine, Tohoku University Graduate School of Medicine, Miyagi, Japan

[10] Department of Integrative Genomics, Tohoku Medical Megabank Organization, Tohoku University, Miyagi, Japan

[11] Department of Human Genetics, Graduate School of Medicine, The University of Tokyo, Tokyo, Japan

[12] Graduate School of Information Sciences, Tohoku University, Miyagi, Japan

[13] Department of Retinal Disease Control, Tohoku University Graduate School of Medicine, Miyagi, Japan

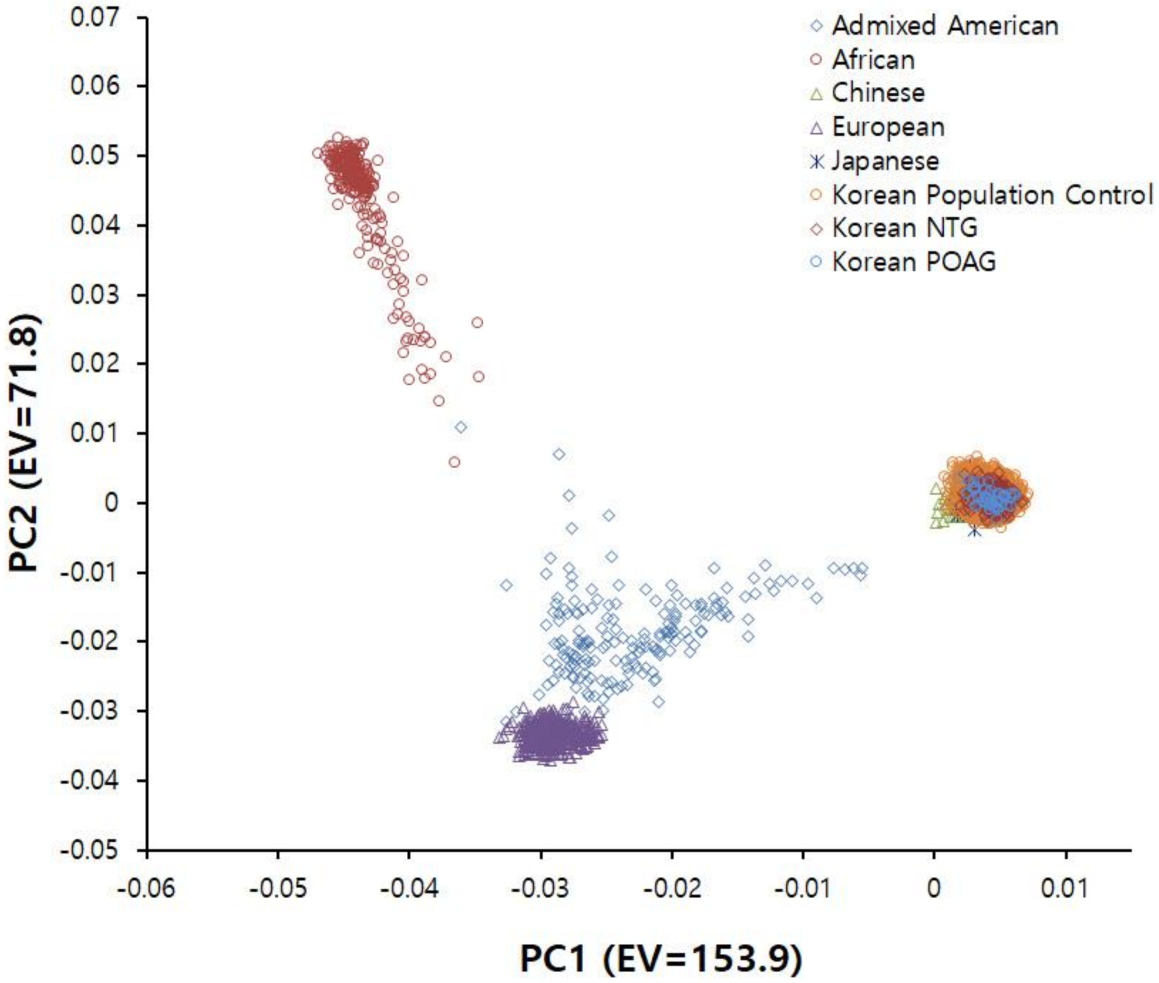

Supplementary Figure S1. Principal component analysis of study participants. First and second principal components of our study samples and 1000 genomes project samples.

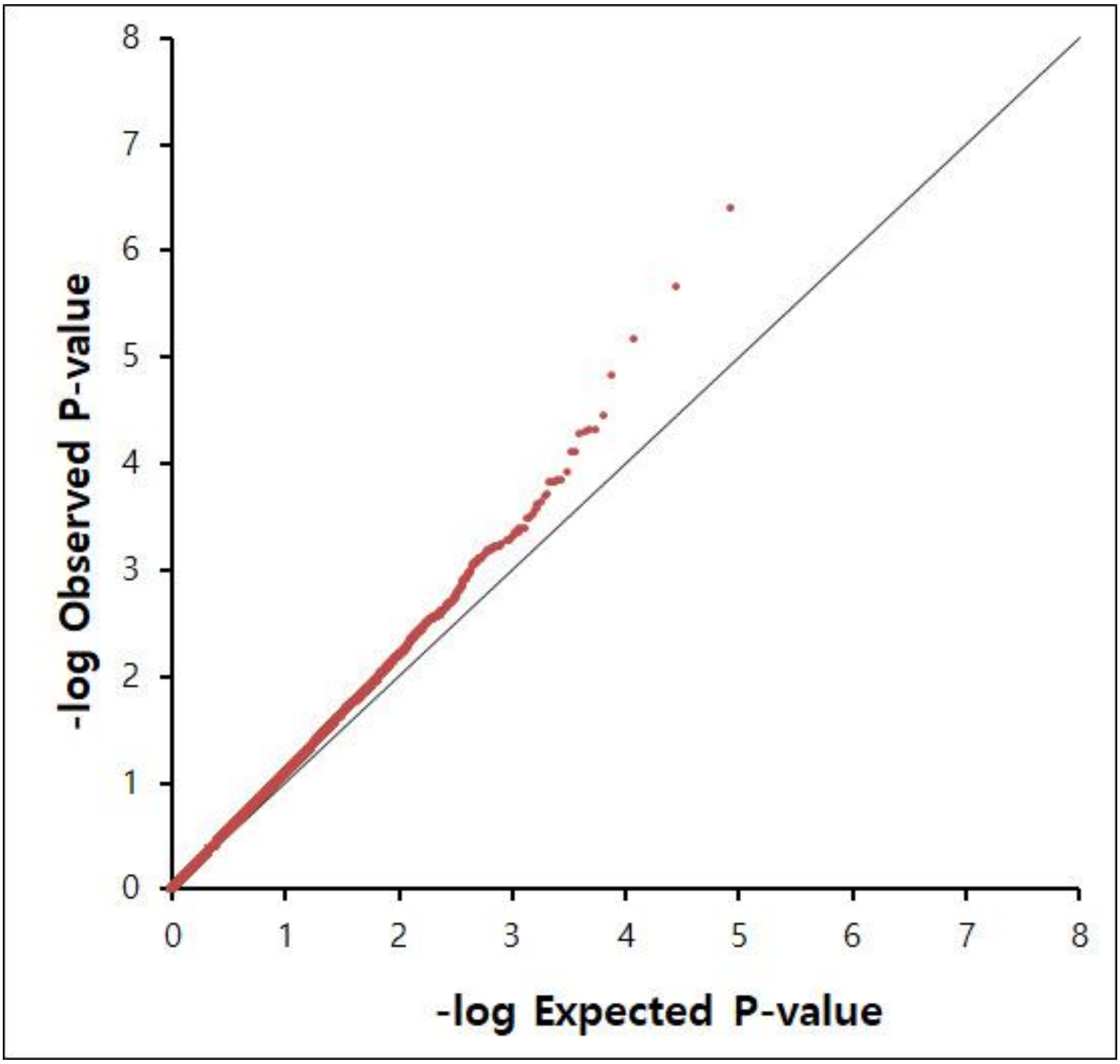

Supplementary Figure S2. Quantile-quantile plots of *P* values in the association results of the exome chip analysis on the Korean population. Under the null hypothesis, the plots would be expected to follow the red line (y = x). The genomic inflation factor (lambda) was 1.08, showing no significant dispersion of test statistics from the expected distribution.

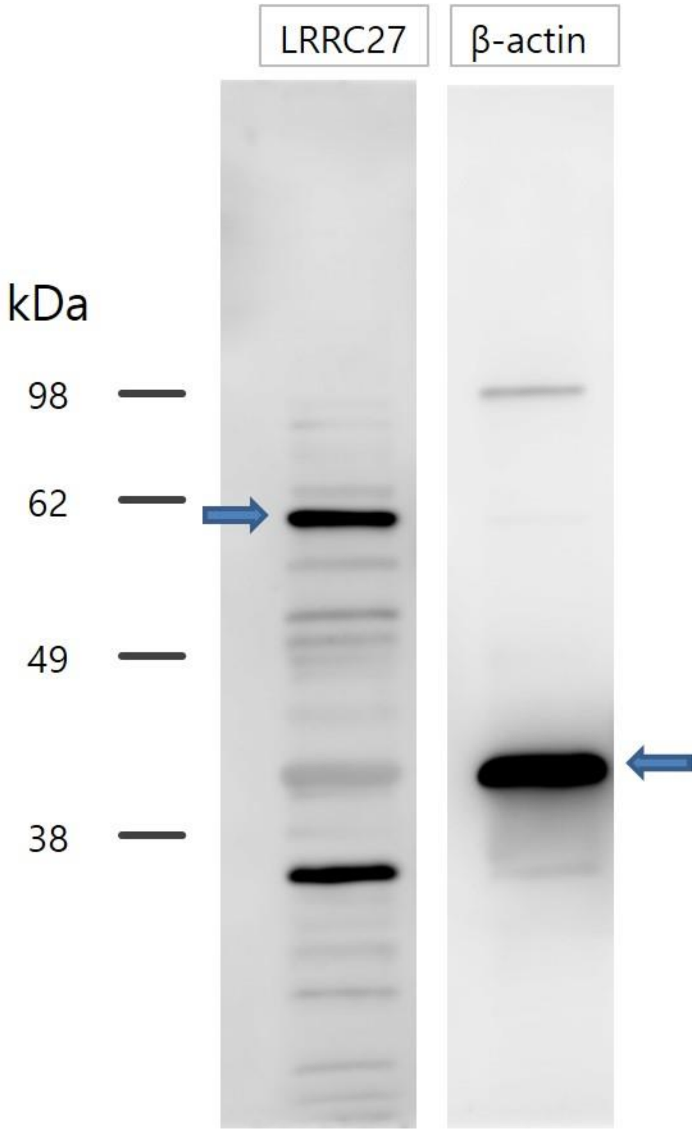

Supplementary Figure S3. Full-length gels and blots for LRRC27 and β-actin.

Supplementary Table S1. Candidate Low-Frequency Variants Associated with Primary Open-Angle Glaucoma at $P < 10^{-4}$

rsID	Gene	Chr	Position	AA change	Alleles	Stage	MAF Case	MAF Control	OR (95% CI)	*P*-value
rs138980799	IVL	1	152882758	H162R	A>G	**Primary (KOR)**	**0.00971**	**0.00009**	**83.24 (9.74–711.44)**	**4.0E-07**
						Replication#1 (KOR)	0.00000	0.00018	NA	NA
						Replication#2 (JPN)	0.00000	0.00000	NA	NA
						**Combined (KOR)**	**0.00485**	**0.00014**	**27.40 (6.70–111.97)**	**1.6E-05***
						Meta-analysis (KOR+JPN)			NA	NA
rs191590289	METTL20	12	31820716	D194V	A>T	**Primary (KOR)**	**0.03560**	**0.01048**	**3.42 (2.13–5.48)**	**6.9E-06**
						Replication#1 (KOR)	0.00485	0.01186	0.41 (0.13–1.31)	0.08
						Replication#2 (JPN)	0.00000	0.00000	NA	NA
						**Combined (KOR)**	**0.02023**	**0.01119**	**1.83(1.21–2.78)**	**0.012***
						Meta-analysis (KOR+JPN)			NA	NA
rs140732889	ZNF677	19	53741442	G180R	C>T	**Primary (KOR)**	**0.00809**	**0.00037**	**20.72 (5.42–79.27)**	**5.1E-05**
						Replication#1 (KOR)	0.00000	0.00053	NA	NA
						Replication#2 (JPN)	0.00000	0.00000	NA	NA
						**Combined (KOR)**	**0.00406**	**0.00045**	**9.60 (3.22–28.60)**	**0.001***
						Meta-analysis (KOR+JPN)			NA	NA
rs4889261	PKD1L2	16	81213378	L711P	G>A	**Primary (KOR)**	**0.01618**	**0.04630**	**0.33 (0.18-0.63)**	**5.4E-05**
						Replication#1 (KOR)	0.05519	0.04840	1.13 (0.79–1.61)	0.51
						Replication#2 (JPN)	0.03680	0.02959	1.24 (0.83–1.85)	0.29
						Combined (KOR)	0.03566	0.04736	0.73(0.54-1.00)	0.048*
						Meta-analysis (KOR+JPN)			0.89	0.45*
rs13339342	PKD1L2	16	81219187	R636H	C>T	**Primary (KOR)**	**0.01618**	**0.04574**	**0.34 (0.18-0.64)**	**7.8E-05**
						Replication#1 (KOR)	0.05663	0.04758	1.18 (0.83–1.68)	0.36
						Replication#2 (JPN)	0.03680	0.02959	1.24 (0.83–1.85)	0.29
						Combined (KOR)	0.03641	0.04667	0.76(0.56-1.03)	0.07*
						Meta-analysis (KOR+JPN)			0.91	0.45*

Chr: chromosome, AA: amino acid, MAF: minor allele frequency, OR: odds ratio, KOR: Korea, JPN: Japan, NA: not applicable.

***P*-values adjusted by Benjamini-Hochberg method to compensate for multiple comparison.

Supplementary Table S2. Comparison of Associations between Normal-Tension Glaucoma (NTG) and High-Tension Glaucoma (HTG) for Candidate SNPs.

rsID	Gene (near gene)	Stage	MAF Control	NTG			HTG		
				MAF Case	OR (95% CI)	*P*-value	MAF Case	OR (95% CI)	*P*-value
rs138980799	IVL	Primary	**0.00009**	**0.01181**	**101.74 (11.78–878.74)**	**1.5E-07**	0.00000	NA	NA
		Rep#1	0.00018	0.00000	NA	NA	0.00000	NA	NA
		Rep#2	0.00000	0.00000	NA	NA	0.00000	NA	NA
		Combined (KOR)	**0.00014**	**0.00598**	**33.78 (8.24–138.51)**	**1.0E-05**[*]	0.00000	NA	NA
rs191590289	METTL20	Primary	**0.01048**	**0.03346**	**3.16 (1.87-5.37)**	**0.0002**	**0.04545**	**4.85 (1.89–12.45)**	**0.007**
		Rep#1	0.01186	0.00605	0.51 (0.16–1.63)	0.21	0.00000	NA	NA
		Rep#2	0.00000	0.00000	NA	NA	0.00000	NA	NA
		Combined (KOR)	**0.01119**	**0.01992**	**1.81 (1.13–2.88)**	**0.04**[*]	0.02155	2.02 (0.81–4.99)	0.17[*]
rs140732889	ZNF677	Primary	0.00037	0.00197	4.50 (0.48–41.98)	0.26	**0.03636**	**89.07 (21.43–370.22)**	**7.2E-07**
		Rep#1	0.00053	0.00000	NA	NA	0.00000	NA	NA
		Rep#2	0.00000	0.00000	NA	NA	0.00000	NA	NA
		Combined (KOR)	0.00045	0.00100	2.41 (0.30–19.15)	0.46	**0.01724**	**39.93 (12.10–131.73)**	**3.8E-05**[*]
rs4889261	PKD1L2	Primary	**0.04630**	**0.01969**	**0.41 (0.22–0.77)**	**0.002**[*]	0.00000	NA	NA
		Rep#1	0.04840	0.06073	1.25 (0.85–1.83)	0.27	0.03279	0.66 (0.24–1.79)	0.38
		Rep#2	0.02960	0.03514	1.16 (0.72–1.87)	0.54	0.04011	1.37 (0.76-2.49)	0.30
		Combined (KOR)	0.04736	0.03992	0.82 (0.60–1.14)	0.35[*]	**0.01724**	**0.35 (0.13–0.95)**	**0.013**[*]
rs13339342	PKD1L2	Primary	**0.04574**	**0.01969**	**0.42 (0.22-0.79)**	**0.002**	0.00000	NA	NA
		Rep#1	0.04758	0.06250	1.31 (0.90–1.91)	0.17	0.03279	0.67 (0.25–1.82)	0.40
		Rep#2	0.02960	0.03514	1.16 (0.72–1.87)	0.54	0.04011	1.37 (0.76–2.48)	0.3008
		Combined (KOR)	0.04667	0.04083	0.86 (0.62–1.18)	0.41[*]	**0.01724**	**0.36 (0.13–0.96)**	**0.013**[*]

MAF: minor allele frequency, NTG: normal-tension glaucoma, HTG: high-tension glaucoma, OR: odds ratio, Rep: replication.

[*]*P*-values adjusted by Benjamini-Hochberg method to compensate for multiple comparison.

Supplementary Table S3. Gene-based rare variant association analysis using SKAT-O method.

Diagnosis	Gene Name	SNPs	Stage	Glaucoma	Control	*P*-value
POAG	METTL20	N172S, D188N, D194V	**Primary**	**8.41%**	**2.93%**	**0.006***
			Replication#1	2.26%	3.33%	0.38
			**Combined**	**5.49%**	**3.13%**	**0.002**
NTG	METTL20	N172S, D188N, D194V	Primary	7.48%	2.93%	0.69*
			Replication#1	2.41%	3.33%	0.70
			**Combined**	**5.17%**	**3.13%**	**0.02**
HTG	ZNF677	G180R, Y347T	**Primary**	**7.3%**	**0.1%**	**3.6E-05***
			Replication#1	0.00%	0.12%	0.67
			**Combined**	**0.81%**	**0.12%**	**1.5E-06**

POAG: primary open-angle glaucoma, NTG: normal-tension glaucoma, HTG: high-tension glaucoma, SNP: single nucleotide polymorphism. **P*-value adjusted by Bonferroni correction.

Supplementary Table 4. Gender Difference of Minor Allele Frequency for rs116121322 in *LRRC27* in Korean Population

rsID	Gene	Chr	AA change	Alleles	Diagnosis	Gender	Stage	MAF Case	MAF Control	OR (95% CI)	*P*-value
rs116121322	*LRRC27*	10	V189I	G>A	POAG	Male	Primary	0.00649	0.00000	-	-
							Replication#1	0.00588	0.00095	5.69 (1.07–30.32)	0.08
							Combined (KOR)	0.00620	0.00049	13.43 (3.50–51.48)	9.0E–04
						Female	Primary	0.01290	0.00069	15.16 (3.54–64.88_	9.1E–04
							Replication #1	0.00719	0.00017	39.77 (3.46–456.60)	0.004
							Combined (KOR)	0.01020	0.00043	20.59 (6.05–70.10)	1.0E–05

Chr: chromosome, AA: amino acid, MAF: minor allele frequency, OR: odds ratio, POAG: primary open-angle glaucoma, KOR: Korea
